# Role of Trimethylamine N-Oxide in Heart Failure

**DOI:** 10.31083/j.rcm2507240

**Published:** 2024-07-02

**Authors:** Lele Jing, Honghong Zhang, Qiannan Xiang, Huilin Hu, Changlin Zhai, Suining Xu, Hongen Tian

**Affiliations:** ^1^Affiliated Hospital of Jiaxing University: First Hospital of Jiaxing, 314000 Jiaxing, Zhejiang, China; ^2^Department of Cardiology, The First Affiliated Hospital, Xi’an Jiaotong University, 710061 Xi’an, Shaanxi, China

**Keywords:** heart failure, gut microbiota, TMAO, inflammatory response, mitochondrial dysfunction, fecal microbiota transplantation (FMT)

## Abstract

Heart failure (HF) is a clinical syndrome characterizing by typical physical signs and symptomatology resulting from reduced cardiac output and/or intracardiac pressure at rest or under stress due to structural and/or functional abnormalities of the heart. HF is often the final stage of all cardiovascular diseases and a significant risk factor for sudden cardiac arrest, death, and liver or kidney failure. Current pharmacological treatments can only slow the progression and recurrence of HF. With advancing research into the gut microbiome and its metabolites, one such trimethylamine N-oxide (TMAO)—has been implicated in the advancement of HF and is correlated with poor prognosis in patients with HF. However, the precise role of TMAO in HF has not yet been clarified. This review highlights and concludes the available evidence and potential mechanisms associated with HF, with the hope of contributing new insights into the diagnosis and prevention of HF.

## 1. Introduction

Heart failure (HF) has persistently high incidence rates. Epidemiological data show that up to 64 million individuals globally are affected by HF, with the incidence in adults being 1% to 2%, and rising to over 10% in those aged over 70 [1]. HF is caused by a range of factors including myocardial damage, excessive cardiac load, and inadequate ventricular preload. It also has various precipitants, such as respiratory infections, arrhythmias, overloading of volume, and exacerbation of underlying diseases. Presently, HF treatment mainly involves the use of inotropic agents, diuretics, and vasodilators, as well as mitigating precipitating factors to manage the progression and recurrence of HF. Nevertheless, Current treatment methods address only a fraction of the assumed pathophysiological route, and the overall prognosis for HF is poor, with high rates of readmission and mortality. Notably, even in the PARADIGM study, the trial group’s 2-year mortality rate was a significant 20% [2]. Thus, there is an imperative to seek new therapeutic directions by finding novel pathophysiological mechanisms.

## 2. Metabolize of TMAO

### Gut Microbiota

The gut microbiota is a sophisticated microbial complexity that lives in the human digestive tract and plays a crucial role in all physiological and immune processes of the human body [3]. Gut microbiota is composed of bacteria, virus, archaea, fungi and protozoa. Importantly, there are three main bacterial systems of intestinal microorganisms: Firmicutes (including *Clostridia* and *Bacilli*), Bacteroidota (including *Bacteroides*), and Actinobacteriota (including *Bifidobacteria*), with smaller amounts of Lactobacilli, Streptococci, and Escherichia coli [4]. Under normal physiological conditions, the gut microbiota is in a state of equilibrium. Imbalances in gut flora can lead to gut-related diseases such as inflammatory bowel disease, obesity, allergic diseases, diabetes, autism, colorectal cancer and cardiovascular disease. In patients with HF, specific changes in the gut microbiota have been observed [5, 6]. Hayashi *et al*. [7] studied the gut microbiota composition of HF patients compared to a control group matched for age, gender, and comorbidities, to assess differences between the two groups. By sequencing the 16s rRNA gene amplicon, they found that the relative abundance of Bifidobacterium spp. was significantly increased, while that of *Megamonas*, was decreased in hyperlipidemic patients compared to controls [7]. However, Modrego *et al*. [8] showed an increase in *Bifidobacterial* abundance 12 months after a HF episode. Therefore to clarify the role of Bifidobacterium in heart failure, further samples of more in-depth fever studies are needed.

The metabolic products of the gut microbiota play a significant role in the body. For instance, Short-chain fatty acids (SCFA) have anti-inflammatory effects and chemopreventive properties, making them considered as tumor suppressors [9, 10]. One such metabolite, trimethylamine N-oxide (TMAO), has been closely linked to cardiovascular diseases, chronic kidney disease, diabetes, and many other illnesses [11, 12, 13, 14]. Multiple studies have identified plasma TMAO as a predictive factor for cardiovascular disease (CVD) in various cohorts [15, 16, 17]. A meta-analysis showed that for every 10 µmol/L increase in TMAO, the risk of cardiovascular death rises by 7.6% [18], suggesting that elevated plasma TMAO levels signify a higher risk of long-term mortality [19]. Cui *et al*. [20] observed an upregulation of microbial genes regulating TMAO production in patients with HF. A possible mechanism is the impairment of the intestinal mucosal barrier in HF patients, leading to increased permeability and enabling easier translocation of TMAO into the bloodstream, which results in elevated plasma TMAO levels [17]. Other evidence also indicates that higher levels of TMAO in the blood are associated with increased mortality rates in patients with HF [21].

## 3. TMAO

### 3.1 Sources of TMAO

Red meat, eggs, fish, and dairy products are rich in choline, betaine, L-carnitine, phosphatidylcholine, and glycerophosphocholine [22, 23]. These substances are transformed into TMAO through the action of specific gut microbial enzyme systems present in the human intestine. For instance, enzymes like choline TMA lyase (CutC/D), carnitine monooxygenase (CntA/B), betaine reductase, and TMAO reduction enzymes can convert phosphatidylcholine and L-carnitine into TMA [24, 25]. TMA enters the bloodstream through the portal vein and is oxidized in the liver by flavin-containing monooxygenases (FMOs) to form TMAO. In particular, humans convert TMA to TMAO primarily through flavin-containing monooxygenase 3 (FMO3). Studies have found that omnivores, those who consume both meat and plant foods, have higher plasma TMAO levels than vegans or vegetarians [13]. Another study showed that people with a long-term intake of high-fat red meat had higher plasma TMAO levels compared to those on a low-fat or Mediterranean diet [23]. Interestingly, fish flesh, which contains TMAO, has the most significant impact on circulating TMAO levels [26, 27]. Consumption of fish results in a notable increase in TMAO concentrations in both blood and urine. It is important to note that the increase in circulating TMAO after choline supplementation through eggs shows considerable individual variation, suggesting strong interactions between diet and host. A high-fat diet is another factor affecting TMAO concentrations. In healthy men, four consecutive weeks of excessive intake of calories with a high fat content (55%) (>1000 kcal/day) also resulted in an increase in fasting plasma TMAO, which may be related to the presence of choline in the high-fat food consumed [28]. In animal studies, feeding mice choline-rich foods led to elevated circulating levels of TMAO, which in turn led to foam cell aggregation, promoting the formation of atherosclerotic plaques and further cardiovascular disease [17].

### 3.2 TMAO Metabolic Pathways

**Fig. 1.**
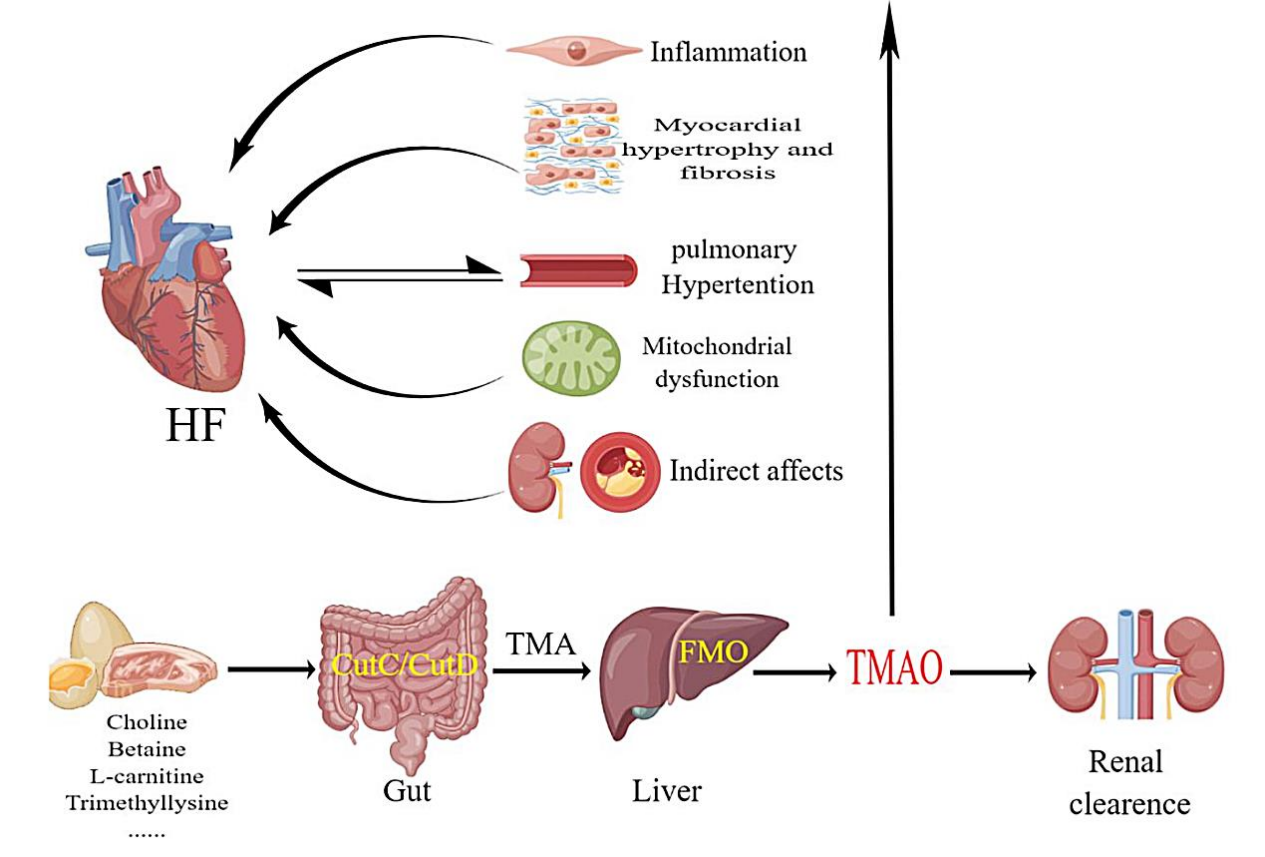
**Humans consume foods rich in choline and carnitine, which are broken down in the intestines by CutC/CutD to produce TMA.** FMO in the liver decomposes TMA into TMAO, which acts on various systems of the body and is eventually excreted by the kidneys. TMAO induces inflammation, fibrosis of cardiomyocytes, pulmonary arterial hypertension, and mitochondrial dysfunction, which can lead to heart failure. FMOs, flavin-containing monooxygenases; CutC/CutD, choline trimethylamine-lyase system; TMA, trimethylamine; TMAO, trimethylamine-N-oxide; FMO, flavinemon-ooxygenase; HF, heart failure.

The dietary-gut microbiota-liver axis forms the biosynthetic pathway for TMAO. Compounds containing a trimethylamine group such as choline, phosphatidylcholine, carnitine, γ-butyrobetaine, betaine, crotonobetaine, and glycerophosphocholine are metabolized by the gut microbiota to produce trimethylamine (TMA). Four main enzymes are involved in the production of TMA: choline TMA cleavage enzyme (CutC/D), carnitine monooxygenase (CntA/B), betaine reductase and TMAO reductase. In addition, the cntA/B homologue yeaW/X also utilizes carnitine, choline, γ-butyricaine, and betaine to produce TMA. [29]. TMA is transported to the liver via the portal circulation and is oxidized by FMO3 to form TMAO [28]. The produced TMAO is distributed to the liver, brain, muscles, kidneys, and intestines, and is involved in various metabolic pathways, exerting either beneficial or detrimental effects on the organism [30]. After consuming choline-rich foods, TMAO levels rise within 4–8 hours, and are then excreted from the body after 24 hours via glomerular filtration or tubular epithelial secretion in the kidneys [31] (Fig. 1).

### 3.3 The Contemporary Research Landscape of TMAO

TMAO was first isolated by researchers in 2013, and since then a growing body of evidence has found that TMAO plays an important role in diseases such as diabetes, chronic kidney disease, cardiovascular disease, and neurological disorders [32, 33, 34]. TMAO was found to induce endoplasmic reticulum (ER) stress in HEK293 cell lines through activation of protein kinase R-like endoplasmic reticulum kinase (PERK) and forkhead box protein O1 (FoxO1), which may lead to insulin resistance, thereby increasing the risk of hyperglycemia, metabolic dysfunction, and type 2 diabetes in individuals with higher levels of TMAO [35]. The effects of TMAO on glucose metabolism have been demonstrated in an animal study. TMAO affects the insulin signaling pathway in the liver, which in turn affects glucose tolerance and increases the synthesis of pro-inflammatory mediators in adipose tissue [36]. There is also evidence that TMAO participates in multiple inflammatory pathways, including Accumulation of lipid deposits in blood vessels and tissues, leading to fatty liver, visceral obesity, and atherosclerosis, and subsequently causing metabolic syndrome [37]. Changes induced by TMAO have also been detected in neurons. *In vitro* models, TMAO generated endoplasmic reticulum stress in hippocampal neurons by activating the PERK pathway, causing alterations in synapse and neuronal plasticity [38]. Animal studies have also shown that TMAO increases clopidogrel resistance to platelets, demonstrating that TMAO can function as a platelet activator [39].

Furthermore, although 95% of TMAO is normally excreted by the kidneys, chronic kidney disease impairs renal filtration. A prospective study evaluating parameters such as glomerular filtration rate, C-reactive protein, and cystatin C found that TMAO was an indicator of survival in patients with chronic kidney disease [40]. TMAO has been reported to accelerate the progression of diabetic nephropathy by activating the NLRP3 inflammasome and promoting the release of IL-1β and IL-18, exacerbating kidney dysfunction and fibrosis [41]. Research has also found that TMAO plays a significant role in regulating blood pressure. On one hand, TMAO is often considered a pathogenic factor in hypertension because it may affect the renin-angiotensin-aldosterone system (RAAS) [42], and enhance the blood-pressure raising effect of angiotensin II (AngII). On the other hand, in hypertensive rat experiments, moderate levels of TMAO were found to reduce diastolic dysfunction and cardiac fibrosis in hypertensive rats, suggesting a potential protective role for myocardial cells under hypertensive conditions [43]. At the same time, a study of 2234 patients with new or worsening HF showed that TMAO levels were significantly associated with adverse outcomes (mortality and rehospitalization), but guideline-based drug therapy did not affect TMAO levels [44]. In summary, future research can focus on TMAO, providing new directions for the treatment and prognosis of various diseases.

### 3.4 TMAO and HF

Recent studies have shown that the gut microbiota and its metabolites, particularly TMAO, play a significant role in the pathophysiology of HF. TMAO has been implicated in the progression of HF and is associated with poor prognosis in patients with this condition. Current research mainly involves the following mechanisms.

## 4. Promotion of Inflammatory Responses

Intramucosal acidosis, reduced ejection fraction, bowel wall thickening and edema have been reported in about 50% of heart failure patients. It is well known that activation of the sympathetic nervous system and the renin-angiotensin-aldosterone system in patients with heart failure is a compensatory response to the reduction in cardiac output and the increase in filling pressures, but ultimately leads to multiorgan underperfusion and circulatory stagnation. In this regard, right heart failure induces intestinal edema, leading to intestinal barrier dysfunction with intestinal leakage of microorganisms and/or their products into the circulation (the “leaky gut” hypothesis of hypertension), as well as low-grade inflammation and associated immune responses [45, 46, 47]. A variety of inflammatory mediators have been found to be elevated in hypertensive patients, including transforming growth factor-β (TGF-β), soluble interleukin (IL)-1 receptor-like 1, tumor necrosis factor-α (TNF-α), soluble TNF receptor type I (sTNFRI), growth differentiation factor 15, IL-6, soluble ST2, and pentraxin-3 [48, 49, 50]. These mediators can promote endothelial dysfunction, myocardial fibrosis, and atherosclerosis by directly activating fibroblasts and recruiting activated macrophages, which in turn promotes myocardial cell necrosis and fibrosis. TMAO up-regulates the expression of these inflammatory mediators, enhance monocyte adhesion, activate NF-κB, and stimulate protein kinase C. These effects may contribute to the progression of chronic HF by increasing endothelial dysfunction, reducing nitric oxide bioavailability, and activating inflammatory responses [51].

### 4.1 NF-κB Signaling Pathway

By activating the nuclear factor kappa-B (NF-κB) pathway, TMAO can induce the expression of inflammatory genes in aortic endothelial cells and vascular smooth muscle cells [52]. Studies in mice *in vivo* and vascular smooth muscle cells *in vitro* have shown that physiological levels of TMAO can induce the expression of cytokines and adhesion molecules. This activation is at least partially mediated by the NF-κB signaling pathway, which is associated with reduced vascular reactivity and inflammation. The mechanism of action of TMAO may be mediated by G protein-coupled receptors (GPCRs), particularly through the activation of the Gßγ subunit complex, which promotes endothelial inflammation, leading to endothelial damage [53]. However, the TMA synthesis inhibitor 3,3-dimethyl-1-butanol (DMB) can prevent cardiac hypertrophy and fibrosis by regulating the NF-κB signaling pathway, confirming the role of TMAO in ventricular remodeling [54].

### 4.2 The NOD-Like Receptor Family, Pyrin Domain Containing 3 (NLRP3) Inflammasome 

NLRP3 inflammasome is a multiprotein complex that plays a central role in inflammatory and autoimmune responses in the body. NLRP3 inflammasome is one of the potential intracellular inflammatory mediators activated during HF. NLRP3 inflammasome promotes the disruption of endothelial tight junctions, leading to endothelial dysfunction and may induce ventricular arrhythmias in patients with atrial fibrillation with a preserved ejection fraction [55, 56]. Conversely, inhibiting the NLRP3 inflammasome can reduce cellular inflammation, hypertrophy, fibrosis, and reverse pathological cardiac remodeling induced by pressure overload [57]. TMAO activates NLRP3 inflammatory vesicles via the SIRT3-SOD2-mtROS (sirtuin-3-superoxide dismutase 2-mitochondrial reactive oxygen species) pathway and induces vascular inflammation [58]. TMAO-induced activation of NLRP3 inflammasomes was found to be associated with redox regulation and lysosomal dysfunction. In animal experiments, direct injection of TMAO into mice with partially ligated carotid arteries resulted in the formation of NLRP3 inflammasomes and increased IL-1β production. Future research should further reveal the specific mechanisms of action between TMAO and the NLRP3 inflammasome and explore the potential for related clinical applications.

### 4.3 PERK

TMAO also exacerbates HF by directly binding to and activating the PERK, which leads to an apoptotic inflammatory response and the production of reactive oxygen species, resulting in vascular damage and cardiac remodeling, which indirectly leads to hypertension [59]. Animal experiments and clinical studies suggest that the activation of TMAO and PERK is closely related to the onset and progression of cardiovascular diseases. Understanding the interaction between TMAO and PERK may provide clues for the development of new drugs for the treatment of HF.

### 4.4 TMAO Promotes Mitochondrial Dysfunction

Makrecka-Kuka *et al*. [60] found that feeding mice 120 mg/kg of TMAO for eight weeks can affect the oxidation of pyruvate and fatty acids, leading to mitochondrial dysfunction and eventually to ventricular remodeling and the development of HF. Additionally, Savi *et al*. [61] consider that TMAO might impact myocardial contractile function through mitochondrial dysfunction. Recent studies involving 200 participants with extreme dietary habits, healthy and unhealthy, showed that the metabolism of the TMAO precursor substance TMA is potentially linked to mitochondrial dynamics and mitochondrial DNA. It promotes the accumulation of mitochondrial reactive oxygen species (mtROS) by inhibiting sirtuin 3 (SIRT3) expression and superoxide dismutase 2 activity and activates NLRP3 inflammatory vesicles to produce IL-1β and IL-18, leading to epithelial cell inflammation [58, 62]. In summary, current research suggests that gut microbiota metabolites may accelerate the progression of HF through promoting mitochondrial dysfunction, with TMAO having a significant impact on mitochondrial function.

### 4.5 TMAO Promotes a Hypercoagulable State

Another mechanism by which TMAO leads to HF is by inducing aortic stiffness, increasing systolic pressure, and causing a hypercoagulable state [63, 64]. Besides affecting coagulation mechanisms, TMAO can also promote the formation of foam cells, induce inflammatory responses, and reduce reverse cholesterol transport, thus promoting atherosclerosis. Although TMAO does not directly cause HF, its impact on blood coagulation states and processes like atherosclerosis indirectly increases the risk of HF, highlighting its significant role in cardiovascular diseases.

### 4.6 TMAO’s Value in HF Risk Stratification and Prognosis

Upon evaluating several studies [15, 16, 17, 18, 19, 20, 21], TMAO has been considered helpful in predicting HF or adverse cardiovascular events. As suggested by the Heianza research group in a paper, TMAO has been identified as a predictor of atherosclerotic coronary artery disease [65]. Moreover, several cohort studies in Europe and the United States have indicated that plasma TMAO concentrations can predict death due to acute myocardial infarction, stroke, or other causes. Independent research has also found that TMAO can be an important predictive factor for HF.

The BIOSTAT-CHF study [44] first explored the reaction of TMAO levels to treatment and discovered that guideline-based CHF drugs may not impact the circulating levels of TMAO. Additionally, patients with lower levels of TMAO had higher survival rates at follow-up. However, patients with persistently rising TMAO levels before and after treatment had a higher mortality rate. Elevated TMAO levels have been shown to correlate with adverse diastolic indices, such as an increased left atrial volume index. A clinical trial examining the effects of TMAO on HF with reduced ejection fraction (HFrEF) and HF with preserved ejection fraction (HFpEF) found that TMAO levels could predict adverse cardiovascular events in patients with HFrEF [66]. Moreover, in a study focusing on HFpEF, TMAO levels proved useful for risk stratification, suggesting that combining BNP with TMAO could provide additional prognostic value for patients with HFpEF [67]. In the future, TMAO may serve as a tool for prognostic prediction and early intervention in HF patients, potentially enhancing patient survival rates.

## 5. Therapeutic Approach

### 5.1 Dietary Control

Diets rich in choline, carnitine, saturated fatty acids, and animal protein have been shown to cause poor development of gut microbiota and elevated plasma levels of TMAO [68]. Levitan *et al*. [69] conducted a retrospective cohort study of 3215 postmenopausal women and showed that patients adhering to the Mediterranean diet had lower urinary TMAO level. Kerley and Nguyen *et al*. [70, 71] found that a plant-based diet rich in fruits, vegetables, legumes, and whole grains may be beneficial for HF patients. Other studies have indicated that reducing the intake of red meat and fats and increasing dietary fiber can decrease plasma TMAO levels and mitigate cardiac remodeling [72, 73, 74]. An analysis of randomized controlled trials (RCTs) has shown that the Mediterranean diet reduces the incidence of HF by up to 70%, suggesting that dietary modifications may reduce plasma TMAO levels [75]. In conclusion, dietary control is an economical and convenient way to prevent and treat HF by positively influencing the gut microbiota.

### 5.2 Antibiotics

Antibiotics can influence diseases caused by microbes by altering the abundance or composition of gut microbiota. Research has shown that antibiotics can suppress the production of TMAO by gut microbiota, thereby reducing the risk of HF progression. Conraads and colleagues found that polymyxin B and tobramycin can reduce the levels of IL-1β, IL-6, and TNF-α in the intestines and feces of patients with HF, improving endothelial function [76, 77]. Participants taking a short course of low-dose antibiotics showed inhibition of TMAO synthesis. Unfortunately, TMAO has been shown to increase the minimum inhibitory concentration of quinolones, aminoglycosides, and β-lactam antibiotics in a concentration-dependent manner and also increased the lethal dose of antibiotics for E. coli [78], suggesting that TMAO can affect the efficacy of antibiotic treatment for HF and is a potential risk factor for the development of antibiotic resistance. While antibiotics have demonstrated some cardiovascular protective effects, this impact seems limited during the medication period. Currently, there is debate about the precise role and effectiveness of antibiotics in improving HF by suppressing TMAO. Further research is needed to verify this role and determine the best treatment strategies and doses. Additionally, misuse of antibiotics can lead to a range of problems, such as resistance and adverse reactions.

### 5.3 Prebiotics and Probiotics

Probiotics play a crucial role in altering the composition of the gut microbiome, maintaining gut homeostasis in the host, and improving human health. Increasing evidence suggests that probiotics may participate in modulating cardiac remodeling in patients with HF. Awoyemi A *et al*. [78] found that *Saccharomyces boulardii* led to an improvement in gut microbiota composition, as well as an improvement in the left ventricular ejection fraction [79]. A 3-month randomized controlled trial (RCT) conducted by Costanza and colleagues revealed that chronic HF patients treated with probiotics exhibited significant reductions in inflammatory markers and improvements in cardiac contractility compared to the control group [80]. Recent studies have indicated that probiotics can decrease TMAO levels, suggesting that their cardioprotective effects might be achieved in part by reducing circulating TMAO [81, 82]. Clinically, the intake of prebiotics has also been proven to be beneficial for patients with cardiovascular diseases. For example, one study showed that oat β-glucan reduced cholesterol, especially low-density lipoprotein (LDL) cholesterol concentrations, in patients with hypercholesterolemia compared to controls. In addition, in the intermittent intestinal fermentation system, oat β-glucan significantly produces short-chain fatty acids, which may be one of the mechanisms by which prebiotics work [83]. Synergistic effects of prebiotics and probiotics have also been tested in patients with coronary artery disease. Administration of Lactobacillus reuteri for 12 weeks resulted in decreased levels of TNF-alpha, high-sensitivity C-reactive protein (hs-CRP), lipopolysaccharides, and scores on the Beck Depression Inventory [84].

### 5.4 FMT

Fecal microbiota transplantation (FMT) involves the transfer of functional microbiota from healthy human feces into the gastrointestinal tract of a patient to re-establish a normally functioning gut microbiota for the treatment of intestinal and extraintestinal diseases. FMT has been shown to alleviate myocarditis in a mouse model of experimental autoimmune myocarditis [85]. Transplantation of gut microbiota from healthy rats to hypertensive rats reduced blood pressure [86]. A study tested the FMT strategy by transplanting feces from healthy blood donors to patients with metabolic syndrome affecting the heart. The results found that patients with higher bacterial diversity improved peripheral insulin sensitivity [87]. Du *et al*. [87] performed 16S ribosomal RNA (rRNA) sequencing on rat fecal samples and showed that TMA levels in feces as well as TMAO levels in the ipsilateral brain and serum were elevated after traumatic brain injury, whereas FMT reduced TMA levels in feces as well as TMAO levels in the ipsilateral brain and serum [88].

Recent studies have shown that more species of pathogenic microorganisms, such as Candida, Campylobacter, Shigella, and Yersinia, can be detected in the feces of patients with HF, and that these microorganisms are associated with the severity of HF [89]. In a randomized double-blind controlled trial involving 20 patients with metabolic syndrome, a single fecal transplant from a vegetarian donor was found to alter the structure of the gut microbiome in some of the patients, but did not affect parameters associated with vasculitis [90]. However, it is currently unclear whether FMT can improve symptoms by reducing TMAO, and further clinical research is needed to answer this question.

## 6. Future Expectations

**Fig. 2.**
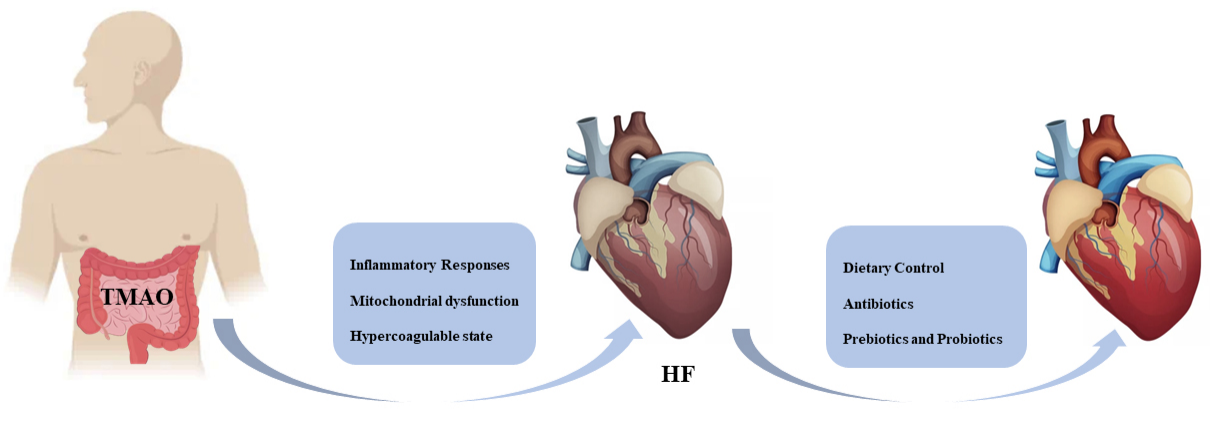
**TMAO precipitates HF by promoting an inflammatory response, mitochondrial dysfunction, and hypercoagulability state.** People can expect to improve heart function through dietary control, judicious use of antibiotics, and taking prebiotics and probiotics. HF, heart failure; TMAO, trimethylamine N-oxide.

In recent years, multiple studies have confirmed the correlation between cardiovascular diseases and gut microbiota as well as the metabolic products of gut microbiota. TMAO, as one of these metabolites, has garnered considerable attention from scholars. Several studies have indicated that TMAO primarily triggers HF through promoting inflammatory responses and mitochondrial dysfunction. However, these studies are fundamentally correlational, and it remains challenging to discern whether changes in TMAO levels are a consequence or cause of the disease. Therapeutically, since TMAO is a metabolite derived from gut microbiota, levels can potentially be reduced through interventions such as diet, fecal transplantation, prebiotics, probiotics, and antibiotics (Fig. 2). Yet, clear empirical evidence is still lacking on whether lowering TMAO levels is effective for the treatment and prognosis of HF. Hence, further prospective studies are necessary to link TMAO to disease onset, progression, and therapeutic measures. Additionally, the role of TMAO in diseases such as hypertension, coronary heart disease, and diabetes should not be overlooked. Meanwhile, beyond TMAO, other gut microbiota metabolites may also possess potential research value. In the future, the modulation of gut microbiota and its metabolic processes may profoundly affect patient survival and prognosis.

## 7. Conclusions

TMAO promotes the progression of HF through inflammatory response, mitochondrial dysfunction and other mechanisms. It may become a new target for HF treatment in the future.
